# NDRG1 Activity in Fat Depots Is Associated With Type 2 Diabetes and Impaired Incretin Profile in Patients With Morbid Obesity

**DOI:** 10.3389/fendo.2021.777589

**Published:** 2021-12-09

**Authors:** Iurii Stafeev, Igor Sklyanik, Elizaveta Mamontova, Svetlana Michurina, Ekaterina Shestakova, Kamil Yah’yaev, Anatoliy Yurasov, Denis Masnikov, Maria Sineokaya, Elizaveta Ratner, Alexander Vorotnikov, Mikhail Menshikov, Yelena Parfyonova, Marina Shestakova

**Affiliations:** ^1^ The Institute of Experimental Cardiology, National Medical Research Center for Cardiology, Moscow, Russia; ^2^ Diabetes Institute, Endocrinology Research Centre, Moscow, Russia; ^3^ Faculty of Basic Medicine, Lomonosov Moscow State University, Moscow, Russia; ^4^ Faculty of Biology, Lomonosov Moscow State University, Moscow, Russia; ^5^ Surgery Department, Central Clinical Hospital #1 of Open Join Stock Company (OJSC) Russian Railways, Moscow, Russia; ^6^ Center of Master’s Programs, I.M. Sechenov First Moscow State Medical University of the Ministry of Health of the Russian Federation (Sechenov University), Moscow, Russia

**Keywords:** omental fat, adipose tissue, insulin resistance, type 2 diabetes, incretins

## Abstract

**Objective:**

We aimed to investigate insulin-, mTOR- and SGK1-dependent signaling basal states in morbidly obese patients’ fat. We analyzed the correlation between the signaling activity, carbohydrate metabolism, and incretin profiles of patients.

**Methods:**

The omental and subcutaneous fat was obtained in patients with obesity. The omental study included 16 patients with normal glucose tolerance (NGT) and 17 patients with type 2 diabetes mellitus (T2DM); the subcutaneous study included 9 NGT patients and 12 T2DM patients. Insulin resistance was evaluated using the hyperinsulinemic euglycemic clamp test and HOMA-IR index. The oral glucose tolerance test (OGTT) for NGT patients and mixed meal tolerance test (MMTT) for T2DM patients were performed. The levels of incretins (GLP-1, GIP, oxyntomodulin) and glucagon were measured during the tests. Signaling was analyzed by Western blotting in adipose tissue biopsies.

**Results:**

We have shown equal levels of basal phosphorylation of insulin- and mTOR-dependent signaling in omental fat depot in NGT and T2DM obese patients. Nevertheless, pNDRG1-T346 was decreased in omental fat of T2DM patients. Correlation analysis has shown an inverse correlation of pNDRG1-T346 in omental fat and diabetic phenotype (HbA1c, impaired incretin profile (AUC GLP-1, glucagon)). Moreover, pNDRG1-T346 in subcutaneous fat correlated with impaired incretin levels among obese patients (inverse correlation with AUC glucagon and AUC GIP).

**Conclusions:**

According to results of the present study, we hypothesize that phosphorylation of pNDRG1-T346 can be related to impairment in incretin hormone processing. pNDRG1-T346 in adipose tissue may serve as a marker of diabetes-associated impairments of the systemic incretin profile and insulin sensitivity.

## 1 Introduction

Currently, overweight, obesity, and type 2 diabetes mellitus (T2DM) are strongly associated with different comorbidities (1, 2). Nevertheless, some patients with obesity can demonstrate a “metabolically healthy” phenotype during a long time without any alterations in carbohydrate metabolism and insulin sensitivity. In this light, the search for new markers and mechanisms of the transition from non-diabetic obesity to severe insulin resistance and T2DM is of much interest to both clinic and basic research (3, 4).

The role of adipose tissue as both the energy buffer and endocrine organ is essential for whole-body nutrient homeostasis. Altered adipocyte insulin sensitivity and glucose utilization lead to an increase in fasting blood glucose and contribute to the development of T2DM (5). Moreover, the heterogeneity and altered distribution of adipose tissue determine metabolic risks. Accumulation of visceral fat is a predictor for threatening metabolic complications such as insulin resistance (6, 7), pro-atherogenic changes in lipid profile (8, 9), and myocardial infarction (10, 11). Therefore, the investigation of visceral (omental) fat metabolism and signaling may hold great potential for understanding T2DM development.

Insulin resistance is a hallmark of obesity-related pathologies that is driven by insulin signaling inactivation (12, 13). The insulin pathway from the activated receptor involves tyrosine phosphorylation of insulin receptor substrate (IRS) and phosphatidylinositol-3-kinase (PI3K) activation leading to phosphorylation of Akt at critical Thr308 and auxiliary Ser473. Akt phosphorylates and inactivates AS160 (Akt substrate with 160 kDa molecular weight, AS160) which inhibits the activity of small Rab GTPases that promote traffic of GLUT4-containing vesicles to the plasma membrane, thus increasing glucose uptake (12–15). mTOR (mechanistic target of rapamycin, mTOR) kinase plays a crucial role in insulin signaling and nutrient sensing. It forms two distinct protein complexes: mTORC1 and mTORC2. While mTORC1 mediates negative feedback in insulin signaling (16), mTORC2 potentiates insulin signaling through permissive phosphorylation of Akt at Ser473 and activation of the *de novo* lipogenesis (17, 18). The adipose-specific knockout of mTORC2 suppresses insulin-stimulated glucose uptake and prevents development of obesity (19).

mTORC2 phosphorylates and activates serum and glucocorticoid-induced kinase type 1 (SGK1) similarly to Akt, thus rendering SGK1-dependent signaling as a reporter of mTORC2 activation (20, 21). SGK1 regulates gene expression through a variety of transcription factors (22, 23). The role of SGK1 in metabolism has been extensively studied. SGK1 affects nutrient homeostasis through upregulation of membrane transporters including GLUT1 and GLUT4 (24, 25). *In vitro* studies suggested that active SGK1 stimulates adipogenesis and insulin resistance (26, 27). Clinical studies also implicated SGK1 activity in adipose tissue inflammation and insulin resistance (28). We have reported that subcutaneous fat SGK1 is associated with the impaired incretin profile of T2DM individuals (29). SGK1 stimulates transcription factors FOXO1 (forkhead box protein O1) and NDRG1 (N-myc downstream-regulated gene type 1) which are involved in white adipogenesis (26, 30). Therefore, the crucial role of SGK1-dependent signaling in T2DM development and progression is highly plausible.

Incretins are the family of peptide hormones produced by intestine L-cells and processed by subsequent proteolysis. The most studied incretins such as glucagon-like peptide type 1 (GLP-1), gastric inhibitory polypeptide (GIP), and oxyntomodulin can stimulate insulin secretion by pancreatic beta-cells and glucose utilization from the blood. Dynamics of the incretin hormone secretion, i.e., the incretin profile, mediate the effects of bariatric surgery’s glucose- and weight-lowering (31). To study the incretin profile interactions with the state of omental fat may help unravel mechanisms of systemic insulin resistance.

Thus, complex interactions between insulin-, mTOR- and SGK1-dependent signalings maintain the general homeostasis of nutrient consumption by adipose tissue. Changes in the adipose tissue signaling state and alterations in paracrine factor secretion may affect distantly on other processes including incretin secretion. In our previous study, we found associations between the basal state of insulin/mTORC2 signaling *via* AS160 and SGK in subcutaneous fat with carbohydrate metabolism and incretin profile (29). However, as we discuss above, omental fat can have more critical roles in developing insulin resistance and this study aims to analyze the insulin- and mTORC2-dependent signaling downstream SGK1 in the omental fat depot, which can be critical to metabolic diseases.

## 2 Methods

### 2.1 Subjects

The study protocol was approved by the ethics committee of the Endocrinology Research Centre (Moscow, Russia) (protocol #9 from 10 May 2017). Written informed consent was obtained from each of the volunteers. Thirty-seven patients with long (>10-year history of overweight) and morbid (BMI > 30 kg/m2) obesity were enrolled in the study; 17 patients had normal glucose tolerance (NGT), and 20 patients had T2DM. Exclusion criteria were age less than 18 years, any other type of diabetes or impaired glucose tolerance, pregnancy, cancer, or inflammation. The NGT patients were not taking any antidiabetic drugs. The T2DM patients in the omental fat group were taking metformin (n = 12), sulfonylurea (n = 5), inhibitors of dipeptidyl peptidase type 4 (n = 7), and inhibitors of sodium-glucose transporter type 2 (n = 4). The T2DM patients in the subcutaneous fat group were taking metformin (n = 10), sulfonylurea (n = 3), inhibitors of dipeptidyl peptidase type 4 (n = 4), and inhibitors of sodium-glucose transporter type 2 (n = 3); 7 patients received monotherapy (by metformin, predominantly), and 13 patients received combined therapy. The omental fat biopsies from 33 patients (16 NGT and 17 T2DM patients) were analyzed. The SGK1 signaling pathway was also validated in subcutaneous fat biopsies from 21 patients (9 NGT patients and 12 T2DM patients). The percentage of sample overlap (patients donated both omental and subcutaneous fat biopsies) was 56.2% for NGT patients and 60% for T2DM patients, respectively.

### 2.2 Glucose Tolerance and Food Load Test

All patients underwent anthropometric measurements (height, weight, hips circumference) and anamnesis collection for durations of obesity, T2DM, and medication usage. The oral glucose tolerance test (OGTT) and mixed meal tolerance test (MMTT) were performed for NGT and T2DM patients, respectively, after an overnight fasting and 12 h of antidiabetic drug deprivation. The blood samples for glucose and incretin measurements were collected before and 30 and 120 min after consuming 82.5 g glucose in OGTT, or Oral Impact mix (Nestle Health Science, Switzerland; 341 kcal, 9.2 g fat, 44.8 g carbohydrates, 18 g proteins) in MMTT.

### 2.3 Insulin Resistance

Systemic insulin resistance was measured by the classic DeFronzo hyperinsulinemic euglycemic clamp test (32) and by HOMA-IR which was calculated as follows:

Insulin resistance = FI* G/22.5

FI - fasting insulin level (uIU/ml)

G - fasting glucose level (mmol/l)

For the clamp test insulin solution, 100 uU/ml was intravenously infused with constitutive rate 1 mU/kg/min using a compact syringe pump. Simultaneously, 20% glucose solution was also infused intravenously to reach normal blood glucose level which was controlled every 5 min using the OneTouch “VerioPro” glucometer (Switzerland). The target blood glucose level was in the range 5.1–5.6 mmol/l. The dynamic equilibrium of the blood glucose level was achieved after 120–180 min of infusion, and at this moment the glucose infusion rate was assumed to be equal to glucose uptake by tissues. When the glucose infusion rate at a dynamic equilibrium and blood glucose level reached the steady state, the M-value was calculated. The results were expressed as M-values (mg/kg/min) and classified into 4 groups of M-values: 0–2 (severe IR), 2–4 (moderate IR), 4–6 (mild IR), and >6 (no IR).

### 2.4 Blood Sample Analysis

HbA1c (reference values 4%–6%) was assessed by high-performance liquid chromatography (D10 Hemoglobin Testing System, Bio-Rad, France). Fasting blood glucose (FBG) (fasting reference values 3.1–6.1 mM) was assessed by ARCHITECT c4000 Clinical Chemistry Analyzer (Abbott Diagnostics, Abbott Park, IL, USA) with manufacturer kits. Immune-reactive insulin was measured in serum with standard kit using electrochemiluminescence analyzer Cobas 6000 (Roche, Switzerland). ELISA kits for adiponectin, leptin, glucagon, and GLP-1 were obtained from Mercodia (Sweden), for GIP—from Cloud-Clone Corp. (USA), and for oxyntomodulin—from Cusabio (USA). The ELISA measurements were performed using 1420 Multilabel Counter VICTOR2 (PerkinElmer, USA).

### 2.5 Body Composition

The amounts of total and visceral fat were assessed by bioelectrical impedance analysis after overnight fasting before OGTT/MMTT using the Body Composition Analyzer Tanita MC-780MA (TANITA Corp., Japan) (33). The analyzer calculates the visceral index (from the 1st to the 55th level) as an estimate of the amount of visceral adipose tissue. The total amount of body fat is shown below as a percentage of total body weight.

### 2.6 Western Blotting

Biopsies from both fat depots were obtained during laparoscopic bariatric surgery (gastric bypass). All fat samples were frozen in liquid nitrogen and stored at -80°C. Biopsies were weighted and homogenized in liquid nitrogen vapor using a porcelain mortar and pestle in radioimmunoprecipitation assay buffer (150 mM NaCl, 1% Triton X-100, 0.5% sodium deoxycholate, 0.1% sodium dodecyl sulfate, 50 mM Tris–HCl, pH 8.0) supplemented with Protease and Phosphatase Inhibitor Cocktail (cOmplete Ultra Tablets, Roche Diagnostics, Germany) at the ratio of 1 μl of buffer per 1 mg of tissue. The extracts were heated for 30 min at 56°C, analyzed by Laemmli polyacrylamide gel supplemented by sodium dodecyl sulfate, and transferred onto polyvinylidene difluoride (PVDF) membranes under 1 A/h. The membranes were blocked by 5% fat-free milk in Tris-buffered saline containing 0.1% Tween 20 (TBST) and incubated overnight with primary antibodies followed (**Supplementary Table 1**) by 1-h incubation with secondary horseradish peroxidase (HRP)-conjugated antibodies (ab6721, Abcam). The protein bands were visualized using Clarity ECL Kit (Bio-Rad, USA) and FUSION FX gel-documentation system (Vilber-Lourmat, France) in the video mode. Quantification of band density was performed using the GelAnalyzer 2010 software.

### 2.7 Statistics

The data were analyzed using SPSS Statistics v.23 software (IBM, USA) and GraphPad Prism 6 (GraphPad Software, USA). Statistically significant differences between NGT and T2DM groups were evaluated by the Mann–Whitney rank-sum U-test. The data are presented as the median and interquartile range. The Spearman correlation was used for correlation analysis. The data are presented as scatter plots with linear trends; p-values < 0.05 are considered significant.

## 3 Results

### 3.1 Comparative Analysis of Clinical Characteristics

General clinical characteristics of patients enrolled in the omental fat study are shown in **Table 1**.

**Table 1:** Characteristics of metabolic parameters and incretin profile of obese patients with (T2DM) and without T2DM (NGT) in the omental fat study groups.

Parameter	Groups		p
	NGT, n = 16	T2DM, n = 17	
**Anthropometric parameters**			
Sex (male:female)	4:12	6:11	N/A
Age, years	44.5 [37.25; 48.5]	46 [36; 54.5]	0.5994
BMI, kg/m^2^	44.9 [41.43; 48.34]	43.25 [39.48; 44.28]	0.0866
Total fat, % of body mass	46.05 [43.65; 48.03]	45.4 [39.35; 48.4]	0.6629
Visceral fat, cm^2^	170 [140; 190]	220 [165; 265.5]	0.009
**Metabolic parameters**			
HOMA-IR	5.12 [3.13; 6.55]	7.82 [6.6; 14.96]	<0.001
M-value, mg/kg/min	3.91 [2.88; 4.72]	1.7 [1.1; 2.16]	<0.001
HbA1c, %	5.6 [5.4; 5.8]	7.6 [6.55; 8.15]	<0.001
Glucose baseline, mmol/L	5.08 [4.95; 5.53]	8.61 [6.87; 9.89]	<0.001
Glucose +120 min, mmol/L	6.06 [4.77; 7.4]	9.78 [7.16; 12.9]	0.001
Insulin baseline, mlU/mL	21.76 [14.5; 26.55]	24.65 [19.44; 35.03]	0.1552
Insulin Δ30–0 min, mlU/mL	128.4 [74.94; 202.5]	44.98 [16.7; 88.86]	0.006
Adiponectin, ng/mL	6.39 [5.95; 7.31]	4.96 [4.25; 5.73]	<0.001
Leptin, ng/mL	36.8 [29.4; 42.95]	35.78 [31.21; 49.03]	0.6928
**Incretin profile**			
GLP-1 baseline, pmol/L	6.98 [5.59; 8.21]	5.19 [4.51; 9.1]	0.7282
GLP-1, Δ30-0 min, pmol/L	29.4 [23; 36.2]	7.67 [6.04; 10.69]	<0.001
GLP-1 120 min, pmol/L	20.28 [13.7; 35.09]	9.73 [8.24; 11.96]	<0.001
AUC GLP-1	61.13 [45.25; 70.11]	22.43 [18.22; 24.5]	<0.001
GIP baseline, pg/mL	587.3 [533.2; 687]	638.6 [599.8; 726.7]	0.028
GIP Δ30–0 min, pg/mL	7.1 [-40; 32.1]	13.21 [-25.65; 24.92]	0.8679
GIP 120 min, pg/mL	598.3 [568.4; 670.2]	620.3 [603.3; 705.7]	0.0625
AUC GIP	1193 [1118; 1359]	1286 [1195; 1429]	0.0935
Oxyntomodulin baseline, pmol/L	0.86 [0.49; 1.54]	0.63 [0.37; 0.77]	0.033
Oxyntomodulin Δ30–0 min, pmol/L	-0.07 [-0.65; 0.19]	-0.1 [-0.27; 0.01]	0.8588
Oxyntomodulin 120 min, pmol/L	0.7 [0.45; 1.26]	0.54 [0.32; 0.83]	0.1875
AUC oxyntomodulin	1.64 [1.18; 2.27]	1.07 [0.7; 1.74]	0.033
Glucagon baseline, pmol/L	3.96 [3.29; 5.09]	8.34 [7.4; 10.04]	<0.001
Glucagon Δ30–0 min, pmol/L	6.77 [6.42; 8.56]	9.71 [8.81; 16.02]	0.003
Glucagon 120 min, pmol/L	6.9 [4.97; 8.52]	11.99 [10.04; 14.65]	<0.001
AUC glucagon	17.27 [14.84; 19.9]	27.93 [22.83; 38.82]	<0.001

The data are shown as a median and interquartile range. p-value <0.05 was considered significant.

N/A, not appropriate.

NGT and T2DM study groups were matched in age, BMI, and total fat values. However, patients with T2DM exhibited higher visceral fat accumulation, consistent with the role of visceral fat in the whole-body metabolic disturbance. The metabolic profile of patients from the T2DM group was prominent: higher basal and after-GTT glucose, higher Hb1Ac, and shifted values of HOMA-IR and M-index to the insulin-resistant state. Adiponectin was suppressed in the T2DM group, whereas leptin level was comparable in NGT and T2DM. Intergroup comparisons of the incretin profile showed impaired secretion of GLP-1 and increased glucagon concentration in T2DM in all time points (the AUC GLP-1 is lower (p < 0.001), and the AUC glucagon is higher (p < 0.001) in the T2DM group, **Table 1** and **Supplementary Figure 1**), except GLP-1 baseline secretion (p = 0.7282). At the same time, GIP and oxyntomodulin displayed only a baseline difference between NGT and T2DM groups (the baseline GIP is higher (p = 0.028) and the baseline oxyntomodulin is lower (p = 0.033) in the T2DM group, **Table 1** and **Supplementary Figure 1**), whereas other timepoints were not significantly different. It should be noted that the major secretion parameter (the area under the curve, AUC) for oxyntomodulin was significantly higher in the NGT group (p = 0.033).

### 3.2 Insulin Signaling in the Omental Fat Is Not Significantly Different in NGT and T2DM Patients

The size of omental fat is highly correlated with insulin resistance according to multiple studies. We therefore asked whether insulin signaling in omental fat is impaired in T2DM. We evaluated phosphorylation of the key components of insulin signaling, i.e., IRS1, Akt, and AS160, the regulator of GLUT4 translocation to plasma membrane. All these values reflect the basal activity of the pathway in the omental fat of patients after overnight fast.

The upstream part of insulin signaling was portrayed by the scaffold protein IRS1 and its activating phosphorylation at tyrosine-612 (pIRS-Y612). The intergroup analysis did not show statistically significant differences between the NGT and T2DM groups (p = 0.163). However, the higher median pIRS1-Y612 in the NGT group is consistent with the literature data (**Supplementary Figures 2A, B** **)**.

Akt kinase is central to receive signals from many pathways. We investigated permissive phosphorylation of Ser473 mediated by mTORC2 (pAkt-S473) and principal for activation Thr308 (pAkt-T308) which is mediated by phosphoinositide-dependent kinase type 1 downstream of PI3K. This analysis of omental fat biopsies did not show differences between NGT and T2DM (p = 0.572 for pAkt-T308 and p = 0.626 for pAkt-S473; **Supplementary Figures 2A, C, D** **)**.

AS160 is the downstream target of insulin signaling that controls translocation of GLUT4-containing vesicles to the plasma membrane (14). We observed no significant difference between NGT and T2DM patients in its phosphorylation on Ser318 (pAS160-S318) (p = 0.654; **Supplementary Figures 2A, E** **)**. For this dataset, we performed correlation analysis with clinical characteristics of patients but it did not reveal any significant correlation. In summary, the basal state of insulin signaling in omental fat of obese patients does not reflect systemic insulin sensitivity.

### 3.3 mTOR Signaling in the Omental Fat Is Equal in NGT and T2DM Individuals

mTOR kinase signaling is implicated in nutrient sensing and regulation of adipose tissue storage function. The two mTOR complexes have different compositions, target proteins, and functions. mTORC1 requires Raptor for assembly and phosphorylates p70 S6 ribosomal kinase (S6K) that reports its activity (34). mTORC2 requires Rictor as scaffold and phosphorylates Akt at Ser-473 and SGK1 which are the markers of mTORC2 activity (21).

A comparative analysis of the intergroup variances has not shown any statistically significant results. Although the pmTOR-S2448 median was moderately increased in the T2DM group (**Supplementary Figures 3A, B** **)**, this phosphorylation is an indirect and debatable reporter of mTOR activity. We conclude that the activities of both mTORC1 (**Supplementary Figures 3A, C, D** **)** and mTORC2 (**Supplementary Figures 3A, E, F** **)** are not significantly different in the omental fat of NGT and T2DM individuals. Moreover, the mTOR-dependent signaling in the omental fat did not correlate with clinical characteristics of the patients.

### 3.4 NDRG1 Is Less Phosphorylated in the Omental Fat of T2DM Patients

In our previous study, we found that phosphorylation of SGK1 in subcutaneous fat correlates with insulin resistance, T2DM, and impaired incretin profile (29). Therefore, we studied in more detail SGK1-dependent signaling in omental fat. We analyzed phosphorylation of SGK1 at Ser422 (pSGK-S422) which is critical for activatory phosphorylation of SGK1 at Thr256 (pSGK-T256). In addition, we probed the SGK1-mediated activatory phosphorylation of Thr346 in NDRG1 transcription factor (30, 35) (pNDRG-T346) in the omental adipose tissues.

**Figure 1:**
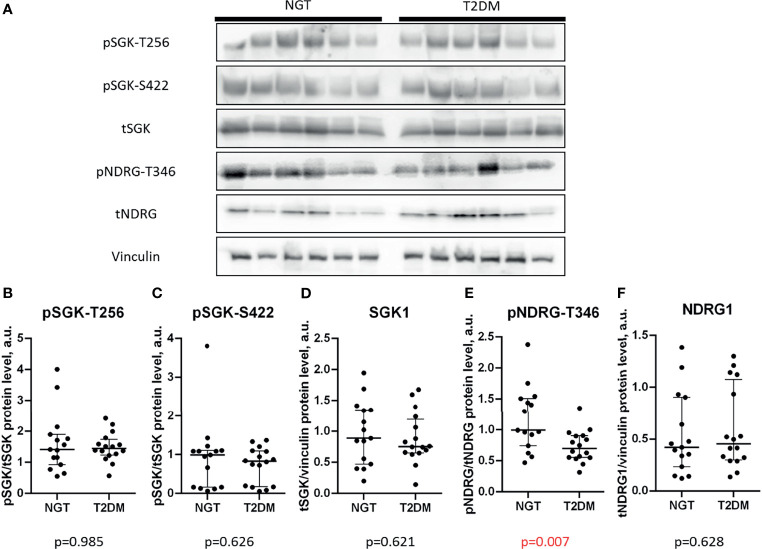
Phosphorylation of NDRG1 is lower in the omental fat of T2DM patients. **(A)** representative Western blots of SGK-dependent signaling; **(B–F)** basal phosphorylation levels and content of SGK1 and NDRG1 in NGT and T2DM obese patients, including pSGK1-T256 **(B)**, pSGK1-S422 **(C)**, total SGK1 **(D)**, pNDRG1-T346 **(E)**, and total NDRG1 **(F)**. The data are shown as the median and interquartile range, n = 31, Mann–Whitney U-test; p values less than 0.05 are considered significant.

The contents of SGK1 and phosphorylated Thr256 or Ser422 were equal in the omental fat of NGT and T2DM subjects (p = 0.985 for pSGK1-T256; p = 0.626 for pSGK1-S422; p = 0.621 for tSGK1; **Figures 1A–D**). However, NDRG1 phosphorylation was significantly lower in T2DM omental fat (p = 0.007; **Figures 1A, E** **)**, whereas expression of NDRG1 was not statistically different in the NGT and T2DM groups (p = 0.628; **Figures 1A, F** **)**. In summary, these data suggest that SGK1 activity in omental fat is unaffected by T2DM under the basal conditions, but activation of its downstream target, NDRG1, is suppressed in T2DM.

### 3.5 NDRG1 Phosphorylation in Omental Fat Correlates With Insulin Resistance and Impaired Incretin Profile

To explore the association of NDRG1 in omental fat with T2DM, we performed a correlation analysis of pNDRG1 and clinical parameters. Hereafter, two “semantic” groups of clinical characteristics, i.e., insulin resistance and incretin profile, were used (29). The correlations with p < 0.05 were considered as statistically significant, 0.05 < p < 0.01—as tendencies.

**Figure 2:**
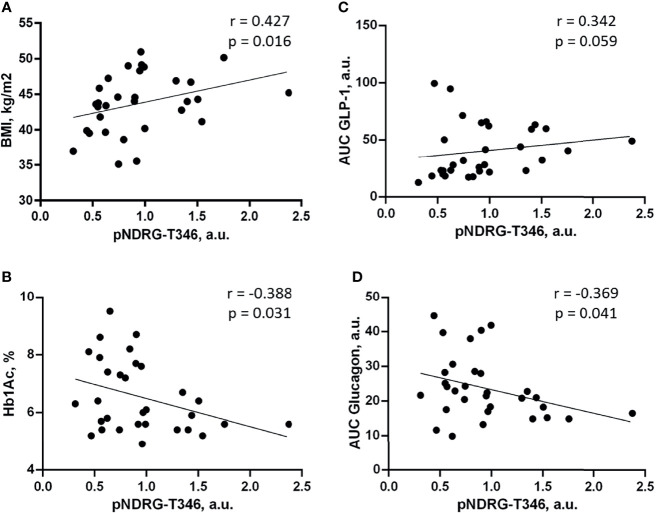
Phosphorylation of NDRG1 at the activatory Thr-346 closely correlates with insulin sensitivity and incretin profile. Shown are scatterplots of the relationships between NDRG1 phosphorylated at T346 (pNDRG1-T346) and BMI **(A)**, Hb1Ac **(B)**, AUC GLP-1 during OGTT/MMTT **(C)**, and AUC glucagon during OGTT/MMTT **(D)**. The linear trends are shown; n = 31; *r*, the Spearman’s correlation coefficient.

We observed a negative correlation between pNDRG-T346 and Hb1Ac blood levels, which is a critical parameter of T2DM diagnosis (p = 0.031; **Figure 2B**). In contrast, pNDRG1-T346 positively correlated with BMI (p = 0.016; **Figure 2A**). In other words, high NDRG1 phosphorylation was associated with higher BMI and lower Hb1Ac. A correlation analysis of incretin secretion profile revealed a positive correlation of pNDRG1-T346 with AUC GLP-1 (p = 0.059; **Figure 2C**) and a negative correlation with AUC glucagon (p = 0.041; **Figure 2D**). Although pNDRG1-T346 was detected in the omental fat of patients with higher BMI, it was associated with “T2DM protective” markers: lower Hb1Ac and glucagon and higher GLP-1. Altogether, these results suggest that phosphorylation of NDRG1 may be a protective response to T2DM, which is impaired in omental fat (see *Discussion* for more details). Thus, pNDRG1-T346 may represent a potential marker of altered carbohydrate metabolism and incretin profile in T2DM.

### 3.6 NDRG1 Content and Phosphorylation in Subcutaneous Fat Also Correlate With Incretin Secretion

The above findings prompted us to analyze the NDRG1 state in subcutaneous fat that has not been done in the previous study (29). The clinical characterization of the study groups is summarized in **Table 2** and **Supplementary Figure 4**; they were comparable in characteristics to those in the omental study above (c.f., **Table 1**). The patients enrolled for the subcutaneous study exhibited diabetes-associated differences in carbohydrate metabolism and incretin secretion profile.

**Table 2:** Characteristics of metabolic parameters and incretin profile of obese patients with and without T2DM included in the subcutaneous fat study.

Parameter	Groups		p
	NGT, n = 9	T2DM, n = 12	
**Anthropometric parameters**			
Sex (male:female)	1:8	5:7	N/A
Age, years	45 [35; 47.5]	42 [36; 52.5]	0.5883
BMI, kg/m^2^	45.84 [44.29; 49.63]	43.25 [40.03; 44.16]	0.009
Total fat, %	45.2 [43.67; 46.91]	48.1 [41.4; 48.4]	0.3838
Visceral fat, cm^2^	160 [145; 190]	240 [185; 270]	0.006
**Metabolic parameters**			
HOMA-IR	4.29 [2.82; 7.12]	6.94 [6.43; 14.96]	0.017
M-value, mg/kg/min	3.95 [2.57; 4.91]	1.44 [0.86; 1.89]	<0.001
HbA1c, %	5.4 [5.3; 5.65]	7.4 [6.55; 8.15]	<0.001
Glucose baseline, mmol/L	5 [4.86; 5.51]	8.71 [6.87; 9.94]	<0.001
Glucose +120 min, mmol/L	6.26 [4.72; 7.72]	9.25 [7.15; 12.07]	0.003
Insulin baseline, mlU/mL	19.78 [13.18; 29.54]	22.42 [16.31; 30.45]	0.4579
Insulin Δ30–0 min, mlU/mL	106.22 [58.14; 165.76]	49.93 [29.32; 74.85]	0.043
Adiponectin, ng/mL	6.65 [5.85; 7.26]	4.94 [4.15; 5.76]	0.002
Leptin, ng/mL	41.77 [27.25; 46.96]	38 [30.34; 49.58]	0.9336
**Incretin profile**			
GLP-1 baseline, pmol/L	6.51 [4.82; 8.15]	5.16 [4.51; 7.96]	0.4277
GLP-1, Δ30–0 min, pmol/L	27.01 [26.27; 34.04]	7.92 [6.32; 7.06]	<0.001
GLP-1 120 min, pmol/L	14.83 [11.97; 28.35]	9.39 [8.24; 11.14]	0.009
AUC GLP-1	50.17 [42.81; 62.97]	22.43 [18.22; 23.13]	<0.001
GIP baseline, pg/mL	569.4 [475.2; 647.2]	683 [605.8; 741.1]	0.005
GIP Δ30–0 min, pg/mL	32.1 [28.4; 52.7]	19.4 [10.2; 21.53]	0.1415
GIP 120 min, pg/mL	594.3 [494.9; 685.1]	694.8 [610.5; 712.5]	0.0596
AUC GIP	1190 [1059; 1379]	1377 [1221; 1441]	0.0507
Oxyntomodulin baseline, pmol/L	0.54 [0.41; 0.81]	0.62 [0.33; 0.77]	0.5436
Oxyntomodulin Δ30–0 min, pmol/L	0.02 [-0.1; 0.24]	-0.03 [-0.19; 0.06]	0.3217
Oxyntomodulin 120 min, pmol/L	0.61 [0.45; 0.95]	0.47 [0.3; 0.81]	0.1779
AUC oxyntomodulin	1.34 [1.04; 1.87]	0.77 [0.61; 1.67]	0.0855
Glucagon baseline, pmol/L	3.81 [3.55; 5.31]	8.34 [7.24; 10.82]	<0.001
Glucagon Δ30–0 min, pmol/L	6.77 [6.37; 8.89]	9.47 [7.64; 11.57]	0.0939 m
Glucagon 120 min, pmol/L	5.13 [4.48; 8.2]	12.62 [10.86; 15.02]	<0.001
AUC glucagon	16.99 [15.02; 20.63]	28.5 [24.22; 40.02]	<0.001

The data are shown as a median and interquartile range. p-value <0.05 was considered significant.

NA, non appropriate.

**Figure 3:**
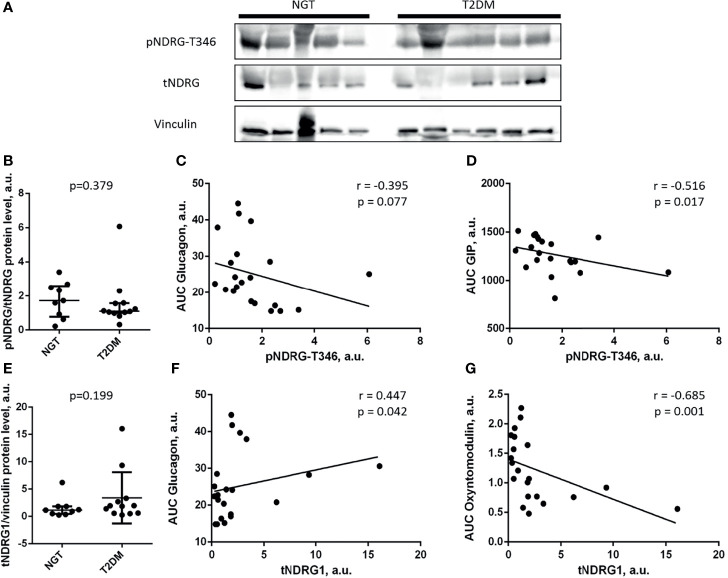
The content and phosphorylation of NDRG1 are equal in subcutaneous fat of NGT and T2DM, but both correlate with incretin secretion. **(A)** Representative Western blots; **(B)** phosphorylation level of NDRG1 at T346; **(C, D)** scatterplots of the pNDRG1-T346 relationship with AUC glucagon **(C)** or AUC GIP **(D)**; **(E)** total NDRG1 content (relative to vinculin); **(F, G)** scatterplots of the total NDRG1 relationship with AUC glucagon **(F)** or AUC oxyntomodulin **(G)**. **(B, E)** The data are given as median and interquartile range, n = 21, Mann–Whitney U-test. **(C, D, F, G)** The linear trends are shown; n = 21; *r*, the Spearman’s correlation coefficient.

In contrast to omental fat, NDRG1 phosphorylation and expression in subcutaneous fat were comparable in the NGT and T2DM groups (p = 0.379 for pNDRG1-T346; p = 0.199 for tNDRG1; **Figures 3A, B, E** **)**. However, a negative correlation between pNDRG1-T346 and AUC glucagon was observed in subcutaneous fat, the same as in omental fat (p = 0.041 for omental fat; p = 0.077 for subcutaneous fat; **Figure 2D**, **Figure 3C**). In contrast to omental fat, pNDRG1-T346 in subcutaneous fat is negatively correlated with GIP (p = 0.017; **Figure 3D**). Interestingly, NDRG1 content directly correlated with AUC glucagon and negatively with oxyntomodulin (p = 0.042 for AUC glucagon; p = 0.001 for AUC oxyntomodulin; **Figures 3F, G** **)**. These results may not be treated as inconsistent: an increase in total NDRG1 content would reduce the pNDRG1/tNDRG1 ratio if T346 phosphorylation is unchanged. In summary, these results suggest a potential role of NDRG1 in both subcutaneous and omental fat depots as a marker of incretin secretion changes and systemic insulin sensitivity.

## 4 Discussion

In this study, we compared the basal state of insulin-, mTOR-, and SGK-dependent signaling in omental fat biopsies of obese patients with or without T2DM and correlated them to metabolic parameters and incretin profile. We find NDRG1 as a marker of impaired incretin secretion and disturbed insulin sensitivity in the omental and subcutaneous fat depots. Possible relationships between SGK1-NDRG1 signaling axis and incretin hormone secretion are discussed below.

### 4.1 Insulin and mTOR Signaling in Omental Adipose Tissue

No differences were found between the NGT and T2DM omental biopsies in basal insulin- and mTOR-dependent signaling. Similar results were obtained in our previous study in subcutaneous fat, where only the pAS160-S318 level was suppressed in T2DM and associated with the impaired incretin profile, whereas other elements of this insulin pathway were not impaired (29). Similarly, no differences between NGT and T2DM groups were observed in mTORC1 or mTORC2 signaling in subcutaneous fat except that only SGK1 phosphorylation at S422 was increased in the T2DM group (29). The most likely explanation is that under the overnight fasting conditions both the insulin and mTOR basal pathways effectively ceased in both omental and subcutaneous fat of the patients. This is consistent with rapid signaling responses to insulin in cells (typically 30–40 min to reach the maximum) and relatively short duration of insulin systemic action (typically few hours in between meals and fully ceased overnight). Arguably, the residual differences displayed by pAS160-S318 and pSGK1-S422 [subcutaneous fat (29)] or by decreased NDRG1 phosphorylation at Thr346 (omental fat, this study, see below) may reflect the altered basal state of insulin/mTOR signaling in T2DM versus NGT subjects. Noteworthily, the basal insulin levels were almost identical after the overnight fast in all studied subjects yet the insulin responses to glucose load (insulin Δ30–0 min) were clearly higher in the NGT groups (29). These may account for increased residual phosphorylations in NGT subjects that persist from the day before the biopsies were taken.

Whether the state of insulin and/or mTOR signaling is already altered (i.e., by obesity) needs further studies compared to healthy lean subjects with normal insulin sensitivity. For example, a clinical study suggested that downregulation of insulin signaling may occur during obesity before T2DM is developed due to hyperactivation of PH domain leucine-rich repeat-containing protein phosphatase (PHLPP-1) (36). Also, a study in a large animal model of insulin resistance has demonstrated absence of correlation between AS160 activity and insulin resistance in either subcutaneous or omental fat depots (37). However, many studies on the expression of insulin signaling participants during obesity and T2DM were carried out. Some of them reported that insulin resistance and obesity are closely associated with enhanced IRS-1 expression (38, 39), and some of them demonstrated opposite results (40, 41). Nonetheless, it should be concluded that most of the differences between insulin signaling of NGT and T2DM lie not in signal but in expression effects. Other clinical studies reported the upregulation of S6K/mTORC1 mRNA and activity in visceral fat depot during insulin resistance (42, 43). On the other hand, mTORC1 signaling was suppressed in primary subcutaneous adipocytes of insulin-resistant patients (44). We suggest that the basal activity of mTOR signaling in omental fat of obese individuals with already altered insulin sensitivity is unlikely strongly associated with further reduction in systemic insulin sensitivity and changes in incretin profile.

### 4.2 NDRG1 Phosphorylation in Omental Fat and Metabolic State

The detailed analysis of the SGK1-dependent signaling pathway in omental fat showed similar levels of SGK1 phosphorylation at S422 and T256 residues in NGT and T2DM. However, we detected a significantly lower NDRG1 phosphorylation at T346 in the T2DM group (p = 0.007). It also positively correlated with BMI and negatively with HbA1c levels (p = 0.016 for BMI and p = 0.031 for Hb1Ac). This may reflect NDRG1 involvement in adipogenesis and glucose metabolism since NDRG1 was found to stimulate adipogenesis in response to phosphorylation by SGK1 (30).

The altered phosphorylation of NDRG1 is of interest because pNDRG1-T346 is taken as a readout of SGK1 and mTORC2 activities (26, 45). However, the content and basal phosphorylation of Akt, SGK1, mTOR, and Rictor were not different between the NGT and T2DM omental fat biopsies (p = 0.572 and 0.626 for Akt; p = 0.626 for SGK1; p = 0.379 for mTOR; p = 0.801 for Rictor). There are a few possible explanations. As noted above, the residual phosphorylation of NDRG1 may persist from the day before being higher in the NGT group owing to higher insulin sensitivity and/or insulin secretion in response to prior food load(s). If so, it would mean that pNDRG1-T346 is impaired in T2DM omental fat possibly due to decreased transient reactivity of mTORC2/SGK1 signaling to lower insulin and a faster return of pNDRG1-T346 to basal level over the night fast. That pNDRG1-T346 is not different between the groups in subcutaneous fat (p=0.379) may reflect the otherwise known different roles of these two fat depots in T2DM. Thus, a positive correlation of pNDRG1-T346 with BMI may reflect the larger impact of subcutaneous fat in BMI of the NGT subjects. In this scenario, increased phosphorylation of NDRG1 seems to be protective from T2DM and is specifically impaired in the omental fat of T2DM subjects consistent with its overall negative correlation with HbA1c levels.

Alternatively, phosphorylation of NDRG1 at T346 can be mTORC2/SGK1-independent and may alter while insulin/mTOR signaling does not. The interactome analysis using the STRING11 database [Homo sapiens genome assembling; high confidence interaction score (>0.7)] identifies 8 possible NDRG1-interacting partners, i.e., SGK1, STK38L, AKT1, MYCN, MYC, NDRG2, TP53, and WISP1. Among these targets, SGK1, AKT1, and p38 kinases are able to drive NDRG1 phosphorylation at T346. In this scenario, p38 kinase activity could be a possible positive regulator of NDRG1 phosphorylation at T346 during obesity. In spite of the absence of NDRG1 interaction with phosphatases by STRING11, dephosphorylation can also take part in the regulation of NDRG1 activity. However, this possibility is based only on the predictions of the interactome database and correlations between clinical parameters, mechanisms of NDRG1 activation, and role in metabolism in adipose tissue remains to be determined.

### 4.3 NDRG1 in Omental and Subcutaneous Fat and Incretin Profile

The correlation of NDRG1 phosphorylation with metabolic parameters inspired us to explore its changes not only in omental but also in subcutaneous fat. Incretin hormones play an important role in metabolic homeostasis and impairment of their secretion closely correlates with diabetic phenotype. In our study, we observed an expected decrease of “antidiabetic” GLP1 and oxyntomodulin and an increase of “pro-diabetic” GIP and glucagon in the T2DM group. In the omental fat, pNDRG1-T346 positively correlated with AUC GLP-1 and negatively with AUC glucagon, whereas in subcutaneous fat it negatively correlated with AUC glucagon and AUC GIP. This may suggest an interconnection between NDRG1 activation in adipose tissue with circulating incretin levels. A correlation analysis of the NDRG1 expression revealed a positive association with AUC glucagon and negative with AUC oxyntomodulin. We believe that this may be partly due to the compensatory increase in NDRG1 expression resulting in the lowered level of NDRG1 phosphorylation at T346.

The significant correlation of NDRG1 phosphorylation and presumably activation in adipose tissue with incretins raises a crucial question as to what is the potential communication mechanism. The incretin hormone concentration in the bloodstream is determined not only by synthesis and secretion rate but also by degradation rate. Dipeptidyl peptidase 4 (DPP4) is the enzyme capable of cleavage and inactivation of incretin hormones GLP-1, GIP, and oxyntomodulin, but not glucagon (46, 47). DPP4 is expressed in both subcutaneous and omental fat as a membrane-associated protein which is cleaved and released into the blood in soluble form. Obesity and insulin resistance are closely associated with increased DPP4 gene expression and soluble DPP4 release (46, 48). The study on thyroid cancer has shown an opposite regulation of NDRG1 and DPP4 expression, suggesting transcriptional control of DPP4 by NDRG1 (49). This is because involvement of pNDRG1-T346 phosphorylation in DPP4 gene expression remains unclear and our suggestion requires further investigations.

In summary, we revealed an association of decreased NDRG1 phosphorylation at the activatory T346 in adipose tissue of T2DM individuals and impaired levels of GLP-1, GIP, and oxyntomodulin. Further experiments will be needed to determine how NDRG1 is activated and how the adipose NDRG1 regulates the blood levels of incretin hormones.

## 5 Limitations

Blood sampling during OGTT and MMTT was performed separately from bariatric surgery and acquiring adipose tissue biopsies. However, the preliminary care was equal for both the tolerance tests and surgery including overnight fasting and drug withdrawal before manipulations. Quantification of Western blot band density has high variation levels, and it is also one of the limitations for the correlation analysis with clinical parameters. The correlation analysis was performed for all patients combined from NGT and T2DM groups based on the morbid obesity.

## 6 Conclusions

We conclude that the main finding of the present study is the association between NDRG1 activity and impaired incretin profile. Moreover, this observation is consistent for both subcutaneous and omental fat depots. Our results provide a rationale for future studies on the involvement of NDRG1 in incretin hormone regulation. According to our results, this mechanism can be conservative for different fat depots and work on a systemic level.

## Data Availability Statement

The raw data supporting the conclusions of this article will be made available by the authors, without undue reservation.

## Ethics Statement

The study protocol was approved by the ethics committee of the Endocrinology Research Centre (Moscow, Russia) (protocol #9 from 10 May 2017). The patients/participants provided their written informed consent to participate in this study.

## Author Contributions

ISt collected, analyzed, and interpreted the data and wrote and reviewed the manuscript. ISk performed the preliminary care of patients and collected, analyzed, and interpreted the clinical data. EM and SM collected the data and reviewed the manuscript. KY and AY performed bariatric surgery, obtained fat biopsies, and performed postsurgical care. ES performed the preliminary care of patients and collected and analyzed the data. DM, MSi, MM, and ER analyzed and interpreted the data. AV contributed to analysis of the data and edited and reviewed the manuscript. YP and MSh designed and supervised the study and reviewed the manuscript. All authors contributed to the article and approved the submitted version.

## Funding

This work was supported by the Russian Science Foundation grant #17-15-01435.

## Conflict of Interest

The authors declare that the research was conducted in the absence of any commercial or financial relationships that could be construed as a potential conflict of interest.

## Publisher’s Note

All claims expressed in this article are solely those of the authors and do not necessarily represent those of their affiliated organizations, or those of the publisher, the editors and the reviewers. Any product that may be evaluated in this article, or claim that may be made by its manufacturer, is not guaranteed or endorsed by the publisher.

## Abbreviations

AUC, area under curve; AS160, Akt substrate 160 kDa molecular weight; BMI, body mass index; DPP4, dipeptidyl peptidase type 4; ELISA, enzyme-linked immunosorbent assay; FBG, fasting blood glucose; FOXO1, forkhead box protein O1; GLP-1, glucagon-like protein type 1; GIP, gastric inhibitory polypeptide; GLUT1/4, glucose transporter type 1/4; HOMA-IR, homeostatic model assessment for insulin resistance; HbA1c, glycated hemoglobin; HRP-conjugated antibody, horseradish peroxidase-conjugated antibody; IR, insulin resistance; IRS1, insulin receptor substrate type 1; mTORC1/2, mammalian target of rapamycin complex 1/2; MMTT, mixed meal tolerance test; MYC, MYC proto-oncogene protein; MYCN, N-MYC proto-oncogene protein; NDRG1/2, N-MYC downstream-regulated gene 1/2; NGT, normal glucose tolerance; OGTT, oral glucose tolerance test; PVDF membrane, polyvinylidene fluoride membrane; PHLPP-1, PH-domain and leucine-rich repeat protein phosphatase type 1; RIPA buffer, radioimmunoprecipitation assay buffer; SGK1, serum and glucocorticoid-induced kinase type 1; SDS PAGE, sodium dodecyl sulfate-contained polyacrylamide gel electrophoresis; S6K, p70 ribosomal S6 kinase; STK38L/p38, p38 MAP-kinase; T2DM, type 2 diabetes mellitus; TP53/p53, cellular tumor antigen p53; TBST, Tris buffer solution supplemented by 0.1% Tween 20; WISP1, WNT1-inducible-signaling pathway protein 1.
