# Supplementary material for: NDRG1 Activity in Fat Depots Is Associated With Type 2 Diabetes and Impaired Incretin Profile in Patients With Morbid Obesity
Source: Front Endocrinol (Lausanne). 2021 Dec 9;12:777589. doi: 10.3389/fendo.2021.777589 (PMC8695674; doi:10.3389/fendo.2021.777589)
Supplement: Supplementary file 1 [file DataSheet_1.docx]

# Supplementary material

## Supplementary Table 1. Antibodies used for analysis of the insulin-, mTOR- and SGK1-dependent signaling pathways.

| Antibody | Manufacturer | Catalogue number |
| --- | --- | --- |
| pIRS1-Y612 | Thermo Scientific | 44816 |
| tIRS1 | Cell Signaling | sc3407 |
| pAkt-T308 | Cell Signaling | sc9275 |
| pAkt-S473 | Cell Signaling | sc4060 |
| tAkt | Cell Signaling | sc4691 |
| pAS160-S318 | Cell Signaling | sc8619 |
| tAS160 | Cell Signaling | sc2670 |
| pmTOR-S2448 | Cell Signaling | sc5536 |
| tmTOR | Abcam | ab83495 |
| Raptor | Cell Signaling | sc9771 |
| Rictor | Cell Signaling | sc2114 |
| pS6K-T389 | Cell Signaling | sc9206 |
| tS6K | Cell Signaling | sc9202 |
| pSGK1-S422 | Abcam | ab55281 |
| pSGK1-T256 | Thermo Scientific | 44-1260G |
| tSGK1 | Abcam | ab43606 |
| pNDRG1-T346 | Cell Signaling | sc3217 |
| tNDRG1 | Cell Signaling | sc5196 |
| Vinculin | Abcam | ab18058 |


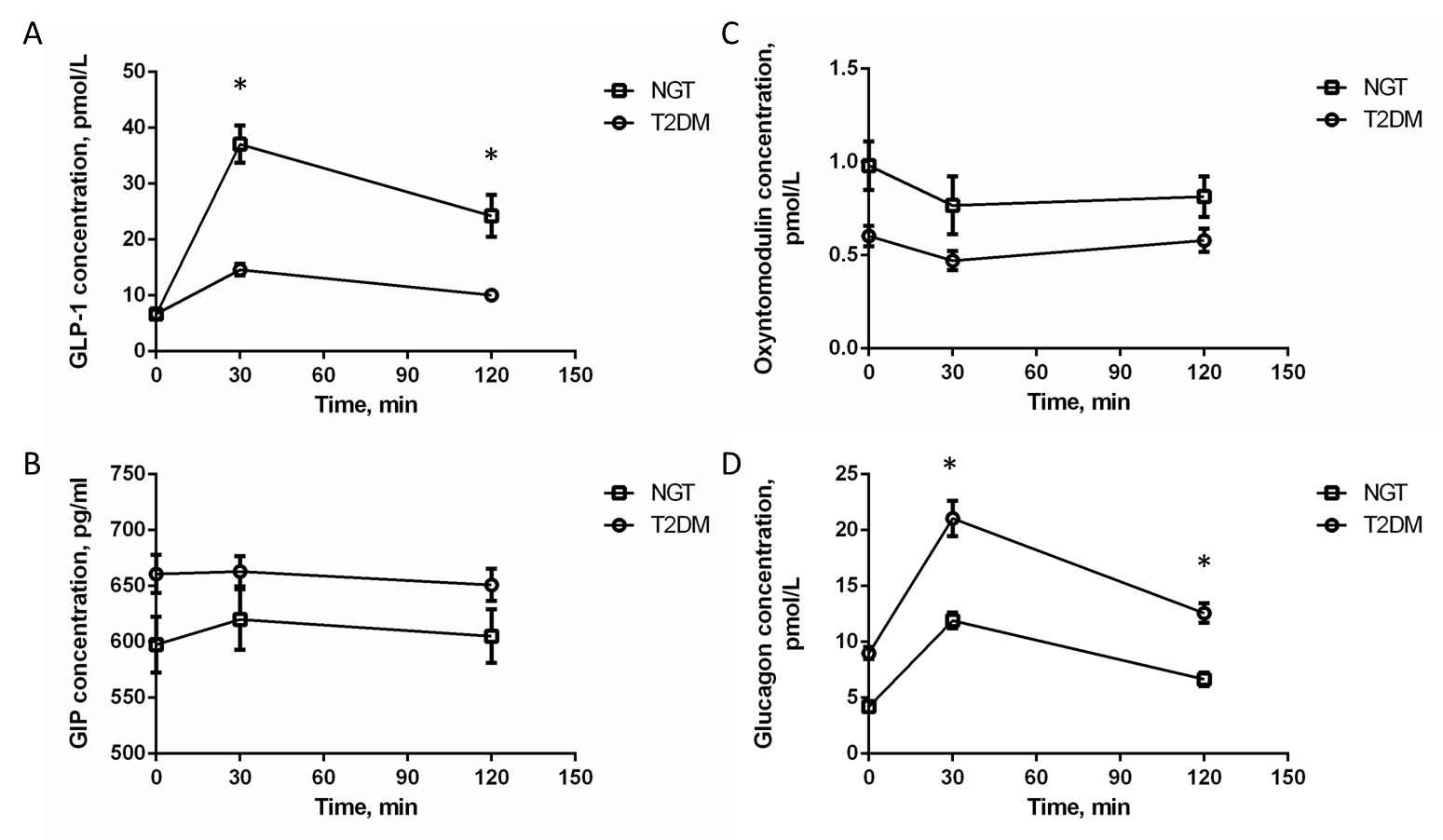


Supplementary Figure 1. Kinetic curves of incretin secretion during OGTT and MMTT for NGT and T2DM patients respectively from omental fat group. A – GLP-1 secretion curve; B – GIP secretion curve; C – oxyntomodulin secretion curve; D – glucagon secretion curve. The data are represented as mean ± SEM; n = 31, Mann-Whitney U-test, * - p<0.05.


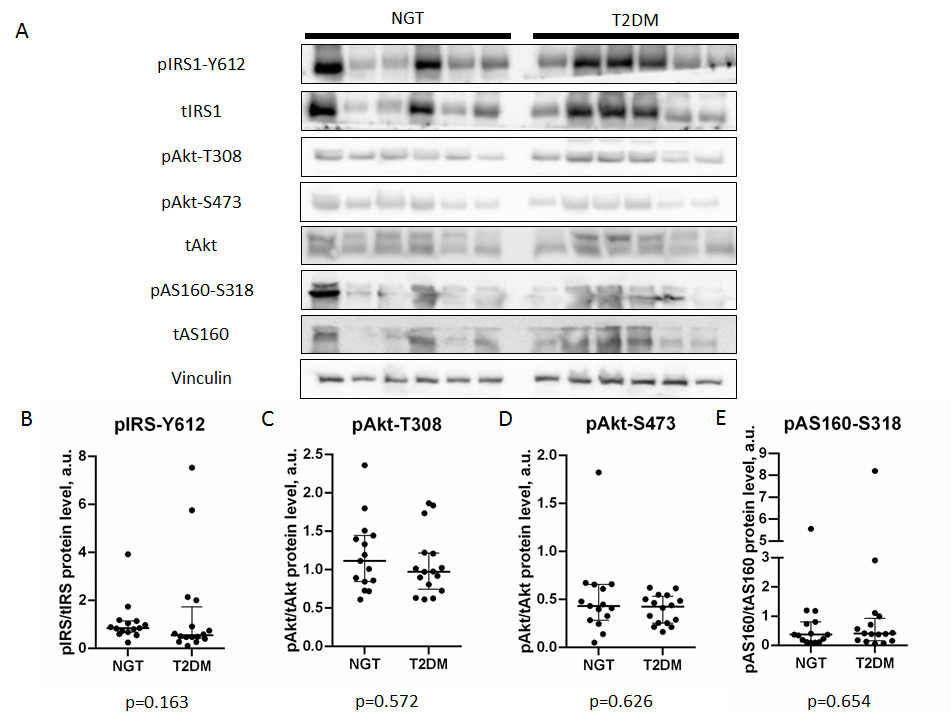


Supplementary Figure 2. Obese patients with and without T2DM display similar basal activity (phosphorylation levels) of insulin pathway compenents in omental fat. A - representative Western blots of insulin-dependent signaling; B-E – intergroup differences of basal phosphorylation levels for insulin-dependent signaling pathway components for NGT and T2DM obese patients: B - pIRS-Y612; C - pAkt-T308; D - pAkt-S473; E - pAS160-S318. The data are shown as the Median and interquartile range, n = 31, Mann-Whitney U-test, p values less than 0.05 are considered significant.


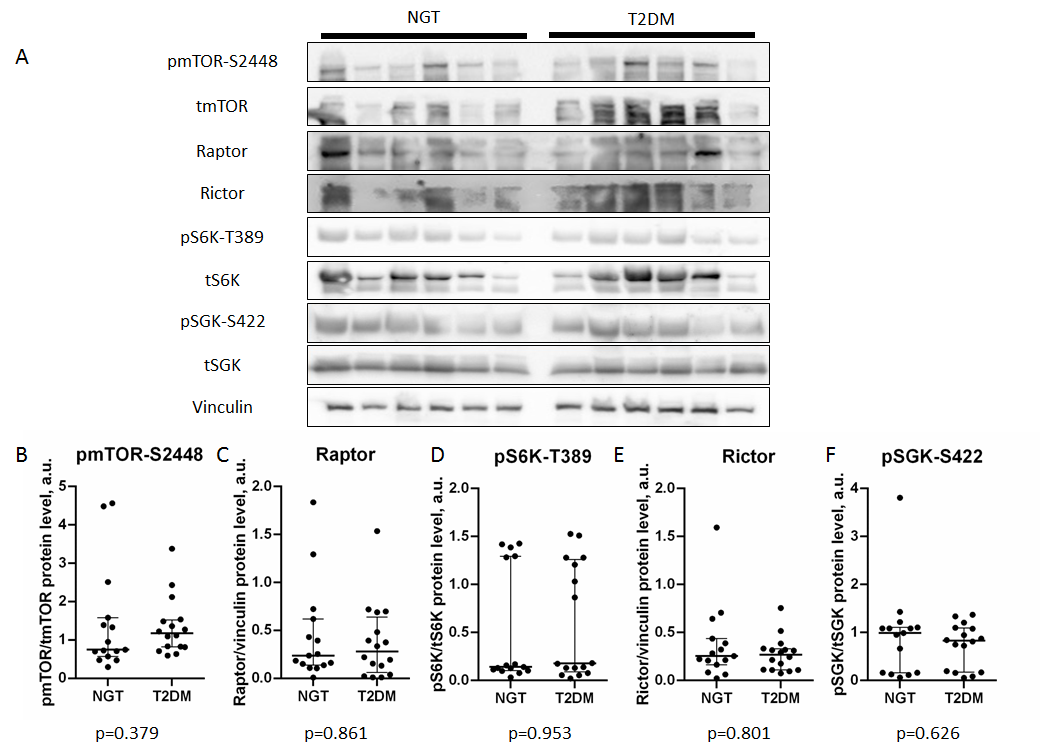


Supplementary Figure 3. Obese patients with and without T2DM display similar basal state of mTOR-dependent signaling pathway in omental fat. A - representative Western blots of mTOR-dependent signaling; B-F - basal phosphorylation levels of the components of mTOR-dependent signaling pathway for NGT and T2DM obese patients: B - pmTOR-S2448; C - Raptor; D - pS6K-T389; E - Rictor; F - pSGK-S422. The data are shown as the Median and interquartile range, n = 31, Mann-Whitney U-test, p values less than 0.05 are considered significant.


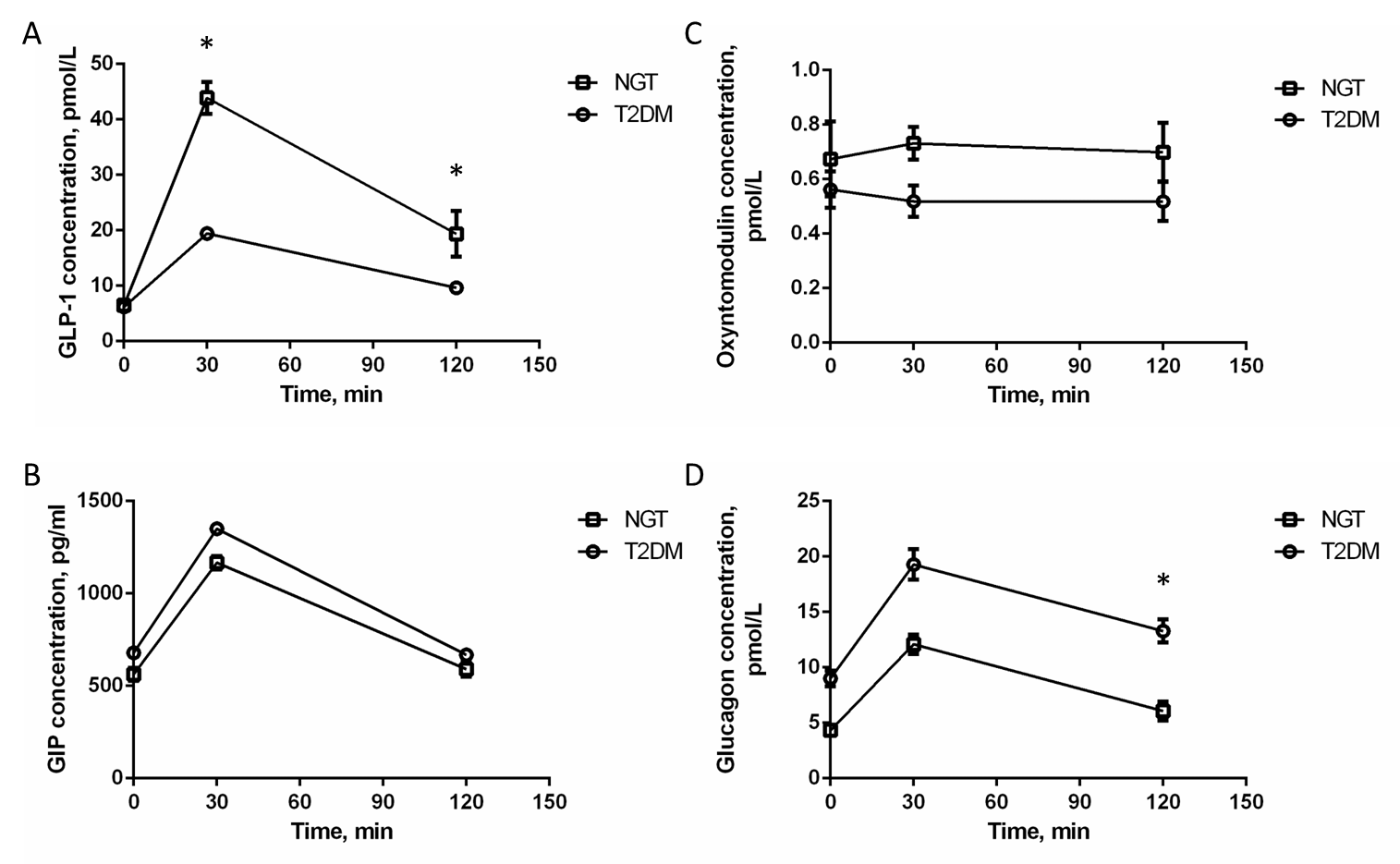


Supplementary Figure 4. Kinetic curves of incretin secretion during OGTT and MMTT for NGT and T2DM patients respectively from subcutaneous fat group. A – GLP-1 secretion curve; B – GIP secretion curve; C – oxyntomodulin secretion curve; D – glucagon secretion curve. The data are represented as mean ± SEM; n = 21, Mann-Whitney U-test, * - p<0.05.
